# Neuroendocrine gene subsets are uniquely dysregulated in prostate adenocarcinoma

**DOI:** 10.1080/15384047.2024.2364433

**Published:** 2024-06-26

**Authors:** Nicole M. Naranjo, Anne Kennedy, Anna Testa, Cecilia E. Verrillo, Adrian D. Altieri, Rhonda Kean, D. Craig Hooper, Jindan Yu, Jonathan Zhao, Oliver Abinader, Maxwell W. Pickles, Adam Hawkins, William K. Kelly, Ramkrishna Mitra, Lucia R. Languino

**Affiliations:** a Prostate Cancer Discovery and Development Program, Thomas Jefferson University, Philadelphia, PA, USA; b Department of Pharmacology, Physiology and Cancer Biology, Thomas Jefferson University, Philadelphia, PA, USA; c Department of Neurological Surgery, Thomas Jefferson University, Philadelphia, PA, USA; d Department of Urology, Emory University School of Medicine, Atlanta, GA, USA; e Department of Human Genetics, Emory University School of Medicine, Atlanta, GA, USA; f Division of Biostatistics and Bioinformatics, Department of Pharmacology, Physiology and Cancer Biology, Thomas Jefferson University, Philadelphia, PA, USA; g Department of Medical Oncology, Thomas Jefferson University, Philadelphia, PA, USA

**Keywords:** Prostate cancer, extracellular vesicles, exosomes, patient plasma, neuroendocrine genes, synaptophysin

## Abstract

Prostate cancer has heterogeneous growth patterns, and its prognosis is the poorest when it progresses to a neuroendocrine phenotype. Using bioinformatic analysis, we evaluated RNA expression of neuroendocrine genes in a panel of five different cancer types: prostate adenocarcinoma, breast cancer, kidney chromophobe, kidney renal clear cell carcinoma and kidney renal papillary cell carcinoma. Our results show that specific neuroendocrine genes are significantly dysregulated in these tumors, suggesting that they play an active role in cancer progression. Among others, synaptophysin (SYP), a conventional neuroendocrine marker, is upregulated in prostate adenocarcinoma (PRAD) and breast cancer (BRCA). Our analysis shows that SYP is enriched in small extracellular vesicles (sEVs) derived from plasma of PRAD patients, but it is absent in sEVs derived from plasma of healthy donors. Similarly, classical sEV markers are enriched in sEVs derived from plasma of prostate cancer patients, but weakly detectable in sEVs derived from plasma of healthy donors. Overall, our results pave the way to explore new strategies to diagnose these diseases based on the neuroendocrine gene expression in patient tumors or plasma sEVs.

## Introduction

Prostate cancer is the most common cancer among men in the United States, with 299,010 estimated new cases diagnosed and 35,250 estimated deaths attributed to the disease this year.^1^ Prostate cancer has heterogeneous growth patterns;^2^ treatment strategies include androgen deprivation therapy.^3^ If the disease has progressed to androgen independence, it becomes castrate-resistant prostate cancer (CRPC);^3^ its prognosis is the poorest when it progresses to neuroendocrine prostate cancer (NEPC) phenotype.^4^

The role of extracellular vesicles (EVs) in cancer has been recently established, as EVs can contribute to cancer progression hallmarks including proliferation, migration, angiogenesis, evasion of cell death, and metastasis.^5^ EVs are heterogeneous, displaying different biogenesis, and size ranges.^6^ small EVs (sEVs) may have an endosomal or non-endosomal origin, and a size range of <200 nm.^6,7^ sEVs are characterized by classical markers such as Syntenin, TSG101, Alix, and the tetraspanins (CD9, CD63, and CD81)^6^. Syntenin, TSG101, and Alix are part of the ESCRT (endosomal sorting complex required for transport) family of proteins, and play important roles in sEV biogenesis, as these proteins regulate multivesicular body formation and the inward budding of intraluminal vesicles.^6^ The tetraspanins are also crucially involved in sEV biogenesis and release as they can re-arrange the membrane curvatures and thus promote sEV budding and facilitate release.^8,9^ sEVs are present in several biological fluids, such as blood (serum or plasma), urine, semen, and milk.^10^ The content of sEVs can differ between cancer and physiological conditions.^11–14^

In this study, we performed a bioinformatic analysis of publicly available RNA-seq data of a neuroendocrine gene panel. This panel included twenty-two genes which were chosen based on the work done by Hofsli et al., who analyzed genes differentially expressed in neuroendocrine and non-neuroendocrine cancer cell lines that did not include prostate cancer cells.^15^ In our study, we used this neuroendocrine gene panel to analyze the differential expression in five different cancer type and normal tissue samples: Prostate adenocarcinoma (PRAD), Breast cancer (BRCA), Kidney Chromophobe (KICH), Kidney renal clear cell (KIRC) carcinoma, and Kidney renal papillary cell (KIRP) carcinoma. BRCA is the most common non-skin cancer in women in the world.^16^ Renal cell carcinoma (RCC) is a relatively rare and heterogeneous group of tumors with distinct mutations, histologic appearance, and clinical behaviors.^17,18^ RCC is frequently divided into KIRC, KIRP, and KICH. KIRC is the most common subtype, and it has been correlated with aggressive clinical features.^17^ KIRP is the second most common RCC subtype, which is also aggressive and has a poor prognosis when metastatic.^19^ KICH is the third most common RCC subtype and is mainly sporadic and indolent with a favorable clinical outcome.^20^ The neuroendocrine gene panel includes conventional neuroendocrine protein markers such as synaptophysin (SYP), chromogranin A (CHGA) and enolase 2,^15^ which are routinely used for the diagnosis of neuroendocrine tumors. SYP is involved in the process of formation, transport, and release of synaptic vesicles and neuronal transmitters.^21^ SYP is a widely used immunohistochemical marker for neuroendocrine tumor diagnosis.^21^ CHGA is a neuroendocrine secretory protein involved in sorting and packaging of neurotransmitters into secretory granules.^22^ CHGA is an important neuroendocrine tumor marker used both in examination of biopsied tumor tissue and as a serum tumor marker. Enolase 2 is an enzyme of the glycolytic pathway and one of the main markers used to diagnose poorly differentiated neuroendocrine tumors.^23,24^ Additionally, we evaluated other neuroendocrine genes that have also been implicated in the pathogenesis of neuroendocrine cancers, such as *MYCN*, GDNF Family Receptor Alpha 2 (*GFRA2*), and the neurofilament protein genes (*NEFL, NEFM, NEFH*).^25–27^

In this manuscript, we show that neuroendocrine genes are expressed in patients diagnosed with PRAD and other cancers such as BRCA, KICH, KIRC, and KIRP. We also provide evidence that SYP and sEV classical markers are detected in PRAD patient plasma-derived sEVs but are barely detectable in healthy donor sEVs.

## Results

### Neuroendocrine genes are differentially expressed across cancer types

**Figure 1.**
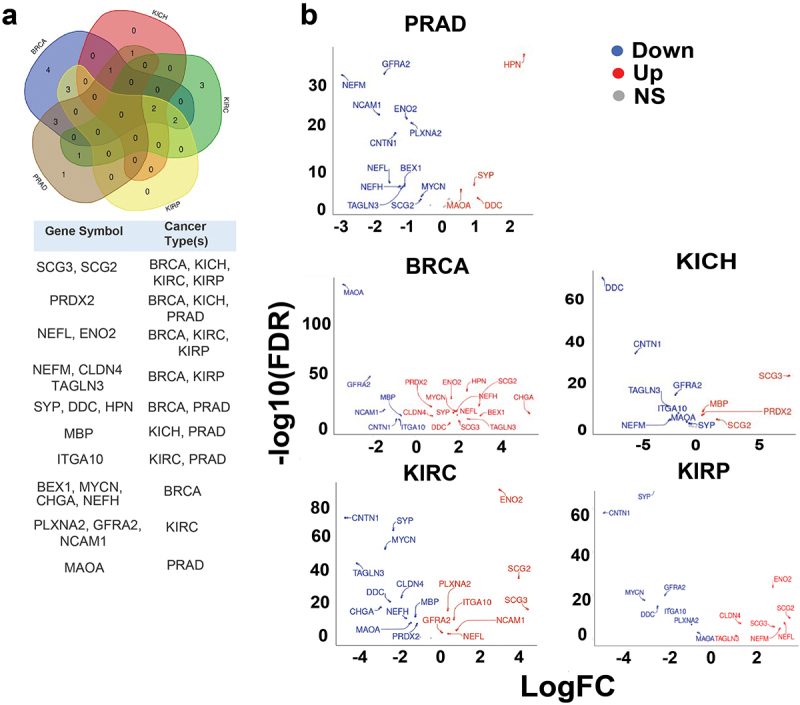
Differential expression patterns of known neuroendocrine cancer genes across TCGA cancer types. (a) Venn diagram depicting the neuroendocrine genes reported in TCGA (The Cancer Genome Atlas) that are significantly upregulated (FDR: False Discovery Rate <0.05) in one or more indicated cancer types [Prostate adenocarcinoma (PRAD), Breast cancer (BRCA), Kidney Chromophobe (KICH), Kidney renal clear cell (KIRC) carcinoma, and Kidney renal papillary cell (KIRP) carcinoma] compared to normal samples. The table below indicates the genes that are significantly upregulated according to the cancer type. (b) Volcano plots of log2 fold-change (Log FC) in expression of the known neuroendocrine genes (X-axis) with statistical significance (Y-axis) in five cancer types. Significantly downregulated and upregulated genes are represented in blue and red colors, respectively. Grey color represents not significant (NS). The genes with 1.5 fold-change are shown. Y-axis represents mRNA expression (Normalized expression). Significantly (FDR <0.05) differentially expressed genes are shown. *P*-value < 0.05 are considered significant.

**Figure 2.**
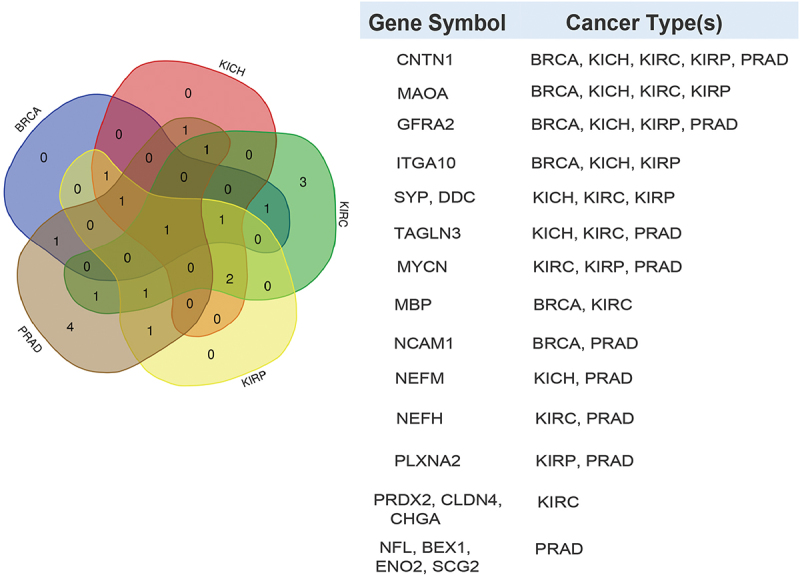
Differential expression patterns of known downregulated neuroendocrine cancer genes across cancer types reported in TCGA. Venn diagram depicting the neuroendocrine genes that are significantly downregulated (FDR <0.05) in different TCGA cancer types (PRAD, BRCA, KICH, KIRC, and KIRP) compared to normal samples. The table (right panel) indicates the genes that are significantly downregulated according to the cancer type.

It has been shown that PRAD patients have distinct heterogeneous molecular alterations that vary between individuals.^2^ Therefore, we decided to analyze the expression levels of literature-curated neuroendocrine genes in PRAD patient tissues compared to normal tissues. In order to broaden the scope of the study, we included in the analysis BRCA, KICH, KIRC, and KIRP patient tissues compared to their healthy counterparts. For this analysis, the RNA-seq expression profiling data were downloaded from the Cancer Genome Atlas (TCGA) database. For TCGA RNA-seq analysis, surgical resection biospecimens were collected from patients diagnosed with PRAD, and had not received prior treatment for their disease (chemotherapy, radiotherapy, or hormonal ablation therapy).^28^ Analysis of TCGA RNA-seq data shows upregulation or downregulation of several genes in cancer versus normal tissues, as shown in Figure 1(a), the volcano plots in Figure 1(b) as well as Figure 2(a). These genes appear to encode proteins found in secretory granules, plasma membrane, cytoskeleton, cytosol, and nucleus (Table 1).

**Table 1.** Dysregulated proteins encoded by neuroendocrine cancer genes.

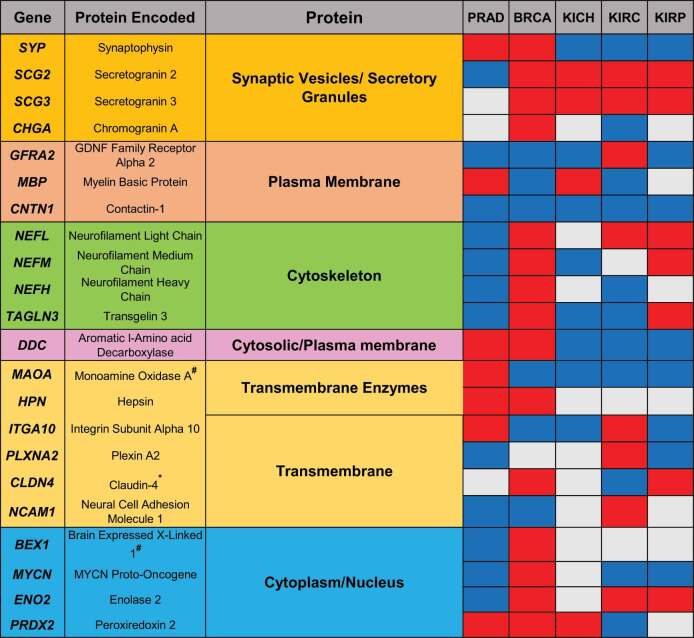

Heat map based on Venn diagrams from Figures 1a and 2.

Red- Upregulated genes.

Blue- Downregulated genes.

Gray- Not significant.

*Putative transmembrane protein.

#Mitochondrial localization.

Proteins related to synaptic vesicles and secretory granules are differentially expressed across cancer types. For instance, *SYP* is upregulated in PRAD and BRCA, and downregulated in KICH, KIRC, and KIRP. Secretogranin 2 (*SCG2*), and Secretogranin 3 (*SCG3*) are the only genes with statistically significant upregulation in four out of the five cancer types compared to the corresponding normal tissue. *SCG2* and *SCG3* are upregulated in BRCA, KICH, KIRC, and KIRP, while *SCG2* is downregulated in PRAD. *CHGA* is upregulated in BRCA and downregulated in KIRC. Furthermore, plasma membrane proteins are dysregulated in different cancers. *GFRA2*, which is significantly upregulated in KIRC, is downregulated in PRAD, BRCA, KICH, and KIRP. Myelin basic protein (*MBP*) is upregulated in PRAD and KICH while being downregulated in BRCA and KIRC. Additionally, Contactin 1 (*CNTN1*) is downregulated in all five cancer types. Of note, the cytoskeletal proteins Neurofilament light chain (*NEFL*), Neurofilament medium chain (*NEFM*), Neurofilament heavy chain (*NEFH*), and Transgelin 3 (*TAGLN3*) are downregulated in PRAD and upregulated in BRCA. In the other cancers, *NEFL* is upregulated in KIRC and KIRP. *NEFM* is upregulated in KIRP and downregulated in KICH. In contrast, *NEFH* is downregulated in KIRC. *TAGLN3* is upregulated in KIRP but is downregulated in KIRC and KICH. The cytosolic protein Dopa decarboxylase (*DDC*) is upregulated in PRAD and BRCA, and downregulated in KICH, KIRC, and KIRP. The transmembrane enzymes Monoamine Oxidase A (*MAOA*), and Hepsin (*HPN*) are upregulated in PRAD. In the other cancers, *MAOA* is downregulated in BRCA, KICH, KIRC, and KIRP, and *HPN* is upregulated in BRCA. Additionally, the other transmembrane proteins are also distinctly dysregulated in different cancers. Integrin alpha subunit 10 (*ITGA10*) is downregulated in BRCA, KICH, and KIRP and upregulated in PRAD and KIRC. Plexin A2 (*PLXNA2*) is downregulated in PRAD and KIRP and upregulated in KIRC. Claudin 4 (*CLDN4*) is upregulated in BRCA and KIRP and downregulated in KIRC. Neural cell adhesion molecule 1 (*NCAM1*) is downregulated in PRAD and BRCA and upregulated in KIRC. Finally, the genes encoding for the cytoplasmic/nuclear proteins, Brain expressed X linked 1 (*BEX1*), *MYCN* and Enolase 2 (*ENO2*) are downregulated in PRAD and upregulated in BRCA. In the other cancers, *MYCN* is downregulated in KIRC and KIRP; *ENO2* is upregulated in KIRC, and KIRP. Peroxiredoxin 2 (*PRDX2*), another protein in this category is upregulated in PRAD, BRCA, and KICH, and is downregulated in KIRC. Collectively, the results show inconsistent dysregulation patterns for most neuroendocrine genes across the cancer types, which is expected due to the high intra-tumor heterogeneity. ^2,29^

**Figure 3.**
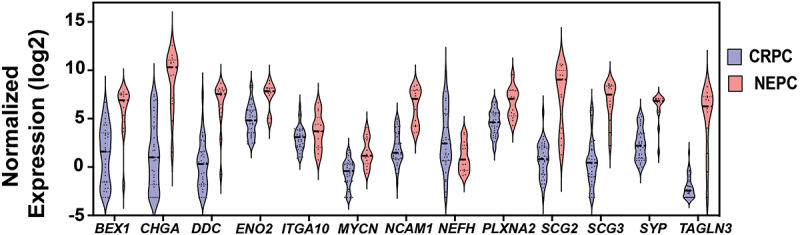
Neuroendocrine gene expression levels in NEPC compared to CRPC. Violin plots represent mRNA expression levels (Y-axis) of neuroendocrine genes (X-axis) that are significantly dysregulated (FDR <0.05) in NEPC compared to CRPC as reported in TCGA. NEPC: neuroendocrine prostate cancer, CRPC: castrate-resistant prostate cancer. *P*-value  < 0.05 are considered significant.

Based on these data, PRAD was selected as an area of specific interest. We then evaluated the levels of these twenty-two genes in CRPC compared to NEPC (Figure 3). These data show that thirteen genes (*SYP, BEX1, CHGA, DDC, ENO2, ITGA10, MYCN, NCAM1, NEFH, PLXNA2, SCG2, SCG3*, and *TAGLN3*) are differentially expressed in NEPC compared to CRPC. Twelve genes are significantly upregulated, and one gene (*NEFH*) is significantly downregulated in NEPC compared to CRPC. As summarized in Table 1, different subgroups of proteins encoded by these genes are significantly dysregulated in PRAD, BRCA, KICH, KIRC or KIRP. Therefore, our data suggest that distinct protein subtypes promote cell differentiation toward a neuroendocrine phenotype in different cancers.

### PRAD patient plasma-derived sEVs express higher levels of synaptophysin and sEV classical markers compared to healthy donor sEVs

**Figure 4.**
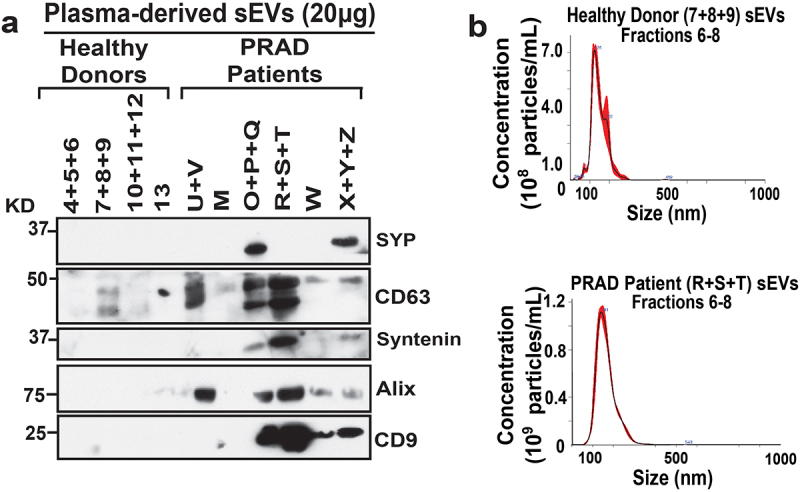
PRAD patient plasma-derived sEVs are enriched in SYP and sEV classical markers. (a) Immunoblotting (IB) analysis of pooled plasma sEV fractions (Fractions 6–8) from healthy donor (4–13) or PRAD patients (M-Z); 20 μg of protein were loaded per sEV sample. Expression of synaptophysin (SYP), CD63, Syntenin, Alix, and CD9 was analyzed. (b) Nanoparticle tracking analysis (NTA) of size and concentration of pooled sEVs (Fractions 6–8) from healthy donor plasma (7 + 8 + 9) or PRAD patient plasma (R+S+T).

Among the genes identified, *SYP* is usually abundant at the protein level in advanced-stage aggressive neuroendocrine in PRAD. Thus, we assessed the levels of SYP in plasma-derived sEVs from PRAD patients and healthy donor plasma sEVs. Furthermore, we also assessed sEV marker levels in plasma sEVs, as it has been shown that sEVs derived from a variety of tumor cells express high levels of sEV classical markers.^30,31^ Immunoblotting (IB) analysis shows that SYP is absent in all healthy donor plasma sEVs but is expressed in two of the PRAD patient plasma sEVs (Figure 4). The IB analysis also shows that sEV markers CD63, Syntenin, Alix, and CD9 are enriched in PRAD patient plasma sEVs compared to healthy donor plasma sEVs (Figure 4a).

Nanoparticle tracking analysis (NTA) shows that the average size of healthy donor and PRAD patient-derived plasma sEVs is ~ 150-160 nm, falling within the expected size range of sEVs (Figure 4b).

We analyzed the plasma-derived sEVs by IB, and our results show that SYP and sEV markers are enriched in patient plasma sEVs. Then, we reviewed groups, which are a five‐tier grade system based on Gleason score grouping and different histology definitions,^32^ the pathological state at diagnosis, and prostate-specific antigen (PSA) levels, and found no correlation with these parameters (Table 2). Patients diagnosed with T2N0M0 (tumor confined within prostate, no regional lymph node metastasis and no distant metastasis)^33^ express different levels of CD63, Syntenin, Alix, and CD9 compared to patients diagnosed with more advanced PRAD, T3BN0M0 (tumor with extracapsular extension-seminal vesicle invasion, with no regional nodal or metastatic spread).^33^ Overall, these data show that SYP and classical sEV marker expression is variable but, in most cases, enriched in PRAD patient plasma sEVs as compared to healthy donor sEVs.

**Table 2.** Synaptophysin and sEV marker expression in PRAD patient derived plasma sEVs.

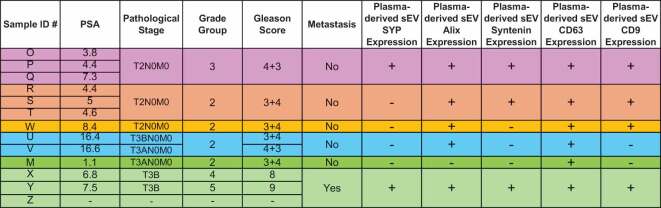

PSA- Prostate specific antigen.

SYP-Synaptophysin.

## Discussion

In this study, we show expression of neuroendocrine genes in PRAD as well as BRCA, KIRC, KICH, and KIRP. Our results pave the way for exploring new perspectives on the functions of neuroendocrine genes in these cancers. Among other genes, we show that *SYP* mRNA levels are increased in PRAD and SYP protein levels are enriched in PRAD patient plasma-derived sEVs, although absent in healthy donor sEVs.

We analyzed RNA-seq data from PRAD, BRCA, KICH, KIRC, and KIRP patient tissues compared to normal tissues to establish a consistent pattern of up- or downregulation of the twenty-two neuroendocrine genes to shed light on the heterogeneity of these genes’ dysregulation patterns across the cancer types. *SYP*, a well-established marker for neuroendocrine cancers,^4,34,35^ is upregulated in PRAD and BRCA, but downregulated in KIRC, KICH and KIRP. Studies have shown that the expression of SYP in PRAD patient tissues is rare since neuroendocrine markers are abundant only when the disease advances to a highly aggressive neuroendocrine stage after androgen deprivation therapy.^4,35,36^ However, our bioinformatic analysis indicates that *SYP* may support the pro-tumorigenic progression of non-neuroendocrine cancers, such as PRAD. Aligning with these results, although some sEVs did not express SYP, it appears that sEVs derived from one PRAD patient with a less aggressive pathological state (T2N0M0), grade group (2) or Gleason score (3 + 4) do not differ in SYP expression compared to plasma sEVs derived from one PRAD patient with more aggressive pathological state (T3BN0M0), grade group (4–5) or Gleason score (8–9). Our data suggest that SYP is highly enriched in sEVs derived from PRAD cells and that these sEVs circulate in the bloodstream. As a result, SYP expression in sEVs derived from PRAD patient plasma sEVs could be instrumental as a prognostic tool for the early detection of PRAD. There are no studies about SYP expression in cancer patient derived EVs. Few studies address the presence of SYP in EVs, but not in the context of cancer.^37,38^ A study investigating the N-glycoproteome of urine-derived EVs (isolated via differential ultracentrifugation) showed that SYP is expressed and N-glycosylated in urine-derived EVs.^37^ SYP has also been shown to be decreased in EVs (isolated by polymer precipitation, ExoQuick) derived from Alzheimer’s disease or frontotemporal dementia compared to healthy controls.^38^ Additional studies screening for SYP in patient plasma-derived sEVs must be investigated in the future.

Furthermore, the discovery that twelve genes have significantly increased expression in NEPC versus CRPC strengthens the relationship of these genes to neuroendocrine transformation and pathogenesis. Overall, our bioinformatic analysis results show that the dysregulation of the neuroendocrine genes in cancer tissues could play a key role in treatment resistance and tumor progression in select cancer types.

Our data show that the classical sEV markers Alix, Syntenin as well as the tetraspanins CD9 and CD63 are enriched in sEVs derived from PRAD patient plasma compared to sEVs derived from healthy donor plasma. This result aligns with our previous studies showing that PRAD patient derived sEVs are enriched in CD9 compared to plasma sEVs from healthy donors.^31^ Furthermore, a study has demonstrated that plasma-derived EVs from pancreatic or lung cancer patients are enriched in classical sEV markers such as CD9, HSPA8, Alix and HSP90AB1 compared to the healthy control counterparts.^30^ Another publication revealed that Alix is enriched in plasma-derived EVs from pancreatic cancer, thus showing its potential to be a biomarker to distinguish early from advanced stage pancreatic cancer.^39^ Similarly, Alix is able to discriminate between plasma-derived EVs from melanoma patients compared to healthy donor plasma EVs.^40^ Moreover, another group has shown that Syntenin is important for increased secretion of sEVs derived from human lung cancer cells, which, in turn, positively regulates endothelial cell migration and tube formation.^41^ Additionally, CD63 has been shown to have increased expression in PRAD patient tissue (grade group 3–5) compared to patients with benign hyperplasia.^42^ Since Alix and Syntenin are part of the molecular machinery responsible for sEV biogenesis,^6^ and the tetraspanins can modulate membrane curvature to enable sEV release,^8^ we can speculate that PRAD patient plasma-derived sEVs are released from prostate cancer cells at a higher degree compared to healthy donor cells and that EV subpopulations may arise as PRAD progresses. These findings synergize with a recently published paper by the Beltran group,^43^ which showed that DNA methylation on specific genomic regions can distinguish NEPC patients from CRPC patients. Thus, they developed a neuroendocrine detection and monitoring (NEMO) assay, in which they used a plasma cell-free DNA-targeted methylation panel that allowed them to stratify and differentiate CRPC from NEPC patients. As a result, it may be possible that cell-free DNA in plasma originates from plasma-derived sEVs, and thus, both represent valuable tools for noninvasive liquid biopsies. Along the same lines, a study showed that BRCA patient plasma-derived EVs or cell-free DNA are both informative biomarker sources in BRCA, specifically for HER2 positive patients.^44^ Moreover, the data demonstrated that combining EV and cell-free DNA assessments increased detection performance.^44^ Therefore, we can speculate that aligning findings from plasma-derived EV content and cell-free DNA from PRAD and NEPC patients may increase the performance of liquid biopsies.

Our results also show that sEVs derived from PRAD patients with a less aggressive form of PRAD (grade group 2) do not significantly differ in sEV marker expression compared to sEVs derived from more advanced (grade group 3–5) PRAD patients; however, the presence of sEV markers vary among them. Therefore, we speculate that sEV marker expression starts in less aggressive forms of PRAD and increases as PRAD progresses. Overall, we provide novel evidence in which a neuroendocrine gene, such as SYP or patient plasma sEV content, can be used as markers for diagnostic purposes in PRAD. Future studies based on proteomic analysis of PRAD and NEPC patient tumors and sEVs will provide a comprehensive understanding of the role of PRAD derived sEVs in promoting cancer progression.

## Materials and methods

### Evaluation of differentially expressed neuroendocrine genes across cancer types

The expression levels of literature-curated neuroendocrine cancer marker genes were evaluated in Prostate adenocarcinoma (PRAD), Breast cancer (BRCA), Kidney chromophobe (KICH), Kidney renal clear cell (KIRC) carcinoma, and Kidney renal papillary cell (KIRP) carcinoma patient samples compared to the normal samples. For this analysis, the RNA-seq expression profiling data were downloaded from the Cancer Genome Atlas (TCGA) database using the R/Bioconductor package TCGAbiolinks.^45^ The read counts were imported to the R package edgeR^46^ to normalize the data and determine differentially expressed genes. Neuroendocrine genes were identified with significant differential expression in: PRAD (*n* = 502) compared to normal (*n* = 52) tissue samples, BRCA (*n* = 1118) compared to normal (*n* = 113) tissue samples, KICH (*n* = 66) compared to normal (*n* = 25) tissue samples, KIRC (*n* = 541) compared to normal (*n* = 72) tissue samples, KIRP (*n* = 290) compared to normal (*n* = 32) tissue samples. The obtained differential expression *P*-values were further adjusted by the Benjamini-Hochberg (BH) method.^47^ Neuroendocrine genes with an adjusted p value < 0. 05 were considered significantly differentially expressed in the respective cancer type compared to its normal samples. Neuroendocrine genes were identified with significant differential expression in neuroendocrine prostate cancer (NEPC) (*n* = 13) compared to PRAD (*n* = 34) samples using the above edgeR analysis pipeline. For this analysis, the RNA-seq expression profiling data were obtained directly from the author upon request.^48^

### Human samples

Blood samples for the isolation of plasma-derived sEVs were obtained from diagnosed PRAD patients (*n* = 13). Biological samples were obtained through a biorepository under an honest broker policy upon patient consent prior to any treatment. Specimens were de-identified in accordance with guidelines established by the Institutional Review Board (IRB), protocol 20D.826. Data collected included pathological stage, Gleason score, grade group, and metastasis at the time of radical prostatectomy; prostate-specific antigen (PSA) was recorded pre-prostatectomy.

Blood samples for the isolation of plasma-derived sEVs were obtained from healthy donors (*n* = 10). Written consent was documented in accordance with the IRB approved protocol 19D.011.

### Plasma isolation

Human blood was withdrawn via venipuncture. Coagulation was prevented by treatment with Acid Citrate Dextrose (ACD) [trisodium citrate (22.0 g/L), citric acid (8.0 g/L) and dextrose (24.5 g/L)]. Anticoagulated blood was centrifuged at 100 *x g* for 20 minutes at room temperature with acceleration 1 and brake 0 to separate plasma.

### sEV isolation

EV isolation by differential ultracentrifugation of healthy donor and diagnosed PRAD patient plasma was performed as previously described.^31,49,50^

sEV isolation from the pellet obtained after ultracentrifugation was further processed by iodixanol density gradient (IDG) ultracentrifugation as previously described.^49–51^ All the data presented in this study were generated using IDG sEVs.

### NTA

NTA was performed as previously described.^50,51^ Briefly, pooled plasma sEVs (fractions 6–8) were diluted in PBS and analyzed using NanoSight NS300. A total of three videos (30 seconds each) were captured. Data were collected at room temperature (25^◦^C). Data analysis was performed using NTA software version 3.1.54.

### IB analysis

IB analysis was performed as previously described.^50–52^ For IB, sEVs and cells were lysed using RIPA buffer (10 mM Tris-HCl, pH 7.4, 150 mM NaCl, 1 mM EDTA, 0.1% SDS, 1% NP-40, and 1% sodium deoxycholate) and protease inhibitors were added. The following primary rabbit Ab was used: Syntenin (Abcam, ab133267). The following primary mouse Abs were used: SYP (Invitrogen MA-1-213), Alix (BIO-RAD, MCA2493), CD63 (Abcam, ab193349), or CD9 (Santa Cruz, sc13118).

The secondary HRP-linked Abs were: anti-rabbit IgG (Cell Signaling, 7074S) or anti-mouse IgG (Cell Signaling, 7076S). For protein visualization, WesternBright^TM^ ECL HRP (horseradish peroxidase) substrate kits (Advansta) were used.

## Data Availability

The data used for the bioinformatics analysis are publicly available. The additional data that support the findings of this study are available from the corresponding authors L.R.L., R.M. or W.K.K. upon reasonable request.

## References

[1] Siegel RL, Giaquinto AN, Jemal A. Cancer statistics, 2024. CA Cancer J Clin. 2024;74(1):12–9. doi:10.3322/caac.21820. 38230766

[2] Haffner MC, Zwart W, Roudier MP, True LD, Nelson WG, Epstein JI, De Marzo AM, Nelson PS, Yegnasubramanian S. Genomic and phenotypic heterogeneity in prostate cancer. Nat Rev Urol. 2021;18(2):79–92. doi:10.1038/s41585-020-00400-w. 33328650 PMC7969494

[3] Beer TM, Armstrong AJ, Rathkopf DE, Loriot Y, Sternberg CN, Higano CS, Iversen P, Bhattacharya S, Carles J, Chowdhury S, et al. Enzalutamide in metastatic prostate cancer before chemotherapy. N Engl J Med. 2014;371(5):424–33. doi:10.1056/NEJMoa1405095. 24881730 PMC4418931

[4] Sainio M, Visakorpi T, Tolonen T, Ilvesaro J, Bova GS. Expression of neuroendocrine differentiation markers in lethal metastatic castration-resistant prostate cancer. Pathol Res Pract. 2018;214(6):848–56. doi:10.1016/j.prp.2018.04.015. 29728311

[5] Moller A, Lobb RJ. The evolving translational potential of small extracellular vesicles in cancer. Nat Rev Cancer. 2020;20(12):697–709. doi:10.1038/s41568-020-00299-w. 32958932

[6] Mathieu M, Martin-Jaular L, Lavieu G, Thery C. Specificities of secretion and uptake of exosomes and other extracellular vesicles for cell-to-cell communication. Nat Cell Biol. 2019;21(1):9–17. doi:10.1038/s41556-018-0250-9. 30602770

[7] Welsh JA, Goberdhan DCI, O’Driscoll L, Buzas EI, Blenkiron C, Bussolati B, Cai H, Di Vizio D, Driedonks TAP, Erdbrügger U, et al. Minimal information for studies of extracellular vesicles (MISEV2023): from basic to advanced approaches. J Extracell Vesicles. 2024;13(2):e12404. doi:10.1002/jev2.12404. 38326288 PMC10850029

[8] Jankovicova J, Secova P, Michalkova K, Antalikova JT. Tetraspanins, more than markers of extracellular vesicles in reproduction. Int J Mol Sci. 2020;21(20):7568. doi:10.3390/ijms21207568. 33066349 PMC7589920

[9] Santos MF, Rappa G, Fontana S, Karbanová J, Aalam F, Tai D, Li Z, Pucci M, Alessandro R, Morimoto C. et al. Anti-human CD9 fab fragment antibody blocks the extracellular vesicle-mediated increase in Malignancy of colon cancer cells. Cells. 2022;11(16):2474. doi:10.3390/cells11162474. 36010551 PMC9406449

[10] Kalluri R, LeBleu VS. The biology, function, and biomedical applications of exosomes. Science. 2020;367(6478). doi:10.1126/science.aau6977. PMC7717626 32029601

[11] DeRita RM, Zerlanko B, Singh A, Lu H, Iozzo RV, Benovic JL, Languino LR. c-Src, insulin-like growth factor I receptor, G-Protein-coupled receptor kinases and focal adhesion kinase are enriched into prostate cancer cell exosomes. J Cell Biochem. 2017;118(1):66–73. doi:10.1002/jcb.25611. 27232975 PMC5552241

[12] Krishn SR, Salem I, Quaglia F, Naranjo NM, Agarwal E, Liu Q, Sarker S, Kopenhaver J, McCue PA, Weinreb PH. et al. The αvβ6 integrin in cancer cell-derived small extracellular vesicles enhances angiogenesis. J Extracell Vesicles. 2020;9(1):1763594. doi:10.1080/20013078.2020.1763594. 32595914 PMC7301698

[13] Osti D, Del Bene M, Rappa G, Santos M, Matafora V, Richichi C, Faletti S, Beznoussenko GV, Mironov A, Bachi A, et al. Clinical significance of extracellular vesicles in plasma from glioblastoma patients. Clin Cancer Res. 2019;25(1):266–76. doi:10.1158/1078-0432.CCR-18-1941. 30287549

[14] Block T, Zezulinski D, Kaplan DE, Lu J, Zanine S, Zhan T, Doria C, Sayeed A. Circulating messenger RNA variants as a potential biomarker for surveillance of hepatocellular carcinoma. Front Oncol. 2022;12:963641. doi:10.3389/fonc.2022.963641. 36582804 PMC9793749

[15] Hofsli E, Wheeler TE, Langaas M, Laegreid A, Thommesen L. Identification of novel neuroendocrine-specific tumour genes. Br J Cancer. 2008;99(8):1330–39. doi:10.1038/sj.bjc.6604565. 18827820 PMC2570516

[16] Waks AG, Winer EP. Breast cancer treatment: a review. JAMA. 2019;321(3):288–300. doi:10.1001/jama.2018.19323. 30667505

[17] Hsieh JJ, Purdue MP, Signoretti S, Swanton C, Albiges L, Schmidinger M, Heng DY, Larkin J, Ficarra V. Renal cell carcinoma. Nat Rev Dis Primers. 2017;3(1):17009. doi:10.1038/nrdp.2017.9. 28276433 PMC5936048

[18] Haake SM, Rathmell WK. Renal cancer subtypes: should we be lumping or splitting for therapeutic decision making? Cancer. 2017;123(2):200–09. doi:10.1002/cncr.30314. 27861752 PMC5222778

[19] Mendhiratta N, Muraki P, Sisk AE Jr., Shuch B. Papillary renal cell carcinoma: review. Urol Oncol. 2021;39(6):327–37. doi:10.1016/j.urolonc.2021.04.013. 34034966

[20] Alaghehbandan R, Przybycin CG, Verkarre V, Mehra R. Chromophobe renal cell carcinoma: novel molecular insights and clinicopathologic updates. Asian J Urol. 2022;9(1):1–11. doi:10.1016/j.ajur.2021.11.010. 35198391 PMC8841285

[21] Stridsberg M. The use of chromogranin, synaptophysin and islet amyloid polypeptide as markers for neuroendocrine tumours. Ups J Med Sci. 1995;100(3):169–99. doi:10.3109/03009739509178905. 8808182

[22] Louthan O. Chromogranin a in physiology and oncology. Folia Biol (Praha). 2011;57(5):173–81. 22123459 10.14712/fb2011057050173

[23] Soh MA, Garrett SH, Somji S, Dunlevy JR, Zhou XD, Sens MA, Bathula CS, Allen C, Sens DA. Arsenic, cadmium and neuron specific enolase (ENO2, γ-enolase) expression in breast cancer. Cancer Cell Int. 2011;11(1):41. doi:10.1186/1475-2867-11-41. 22098917 PMC3233504

[24] Korse CM, Taal BG, Vincent A, van Velthuysen MLF, Baas P, Buning-Kager JCGM, Linders TC, Bonfrer JMG. Choice of tumour markers in patients with neuroendocrine tumours is dependent on the histological grade. A marker study of chromogranin A, neuron specific enolase, progastrin-releasing peptide and cytokeratin fragments. Eur J Cancer. 2012;48(5):662–71. doi:10.1016/j.ejca.2011.08.012. 21945100

[25] Astarloa R, Sanchez-Franco F, Cacicedo L, Garcia-Villanueva M. Differential expression of neurofilament triplet proteins in carcinoid tumours: an immunohistochemical study. Br J Cancer. 1991;63(5):715–18. doi:10.1038/bjc.1991.161. 1903950 PMC1972394

[26] Lee JK, Phillips JW, Smith BA, Park JW, Stoyanova T, McCaffrey EF, Baertsch R, Sokolov A, Meyerowitz JG, Mathis C, et al. N-Myc drives neuroendocrine prostate cancer initiated from human prostate epithelial cells. Cancer Cell. 2016;29(4):536–47. doi:10.1016/j.ccell.2016.03.001. 27050099 PMC4829466

[27] Li Z, Xie J, Fei Y, Gao P, Xie Q, Gao W, Xu Z. GDNF family receptor alpha 2 promotes neuroblastoma cell proliferation by interacting with PTEN. Biochem Biophys Res Commun. 2019;510(3):339–44. doi:10.1016/j.bbrc.2018.12.169. 30722993

[28] Cancer Genome Atlas Research N, Ahn J, Akbani R, Ally A, Amin S, Andry C, Annala M, Aprikian A, Armenia J, Arora A, et al. The molecular taxonomy of primary prostate cancer. Cell. 2015;163(4):1011–25. doi:10.1016/j.cell.2015.10.025. 26544944 PMC4695400

[29] Bowes AL, Tarabichi M, Pillay N, Van Loo P. Leveraging single-cell sequencing to unravel intratumour heterogeneity and tumour evolution in human cancers. J Pathol. 2022;257(4):466–78. doi:10.1002/path.5914. 35438189 PMC9322001

[30] Hoshino A, Kim HS, Bojmar L, Gyan KE, Cioffi M, Hernandez J, Zambirinis CP, Rodrigues G, Molina H, Heissel S, et al. Extracellular Vesicle and particle biomarkers define multiple human cancers. Cell. 2020;182(4):1044–61.e18. doi:10.1016/j.cell.2020.07.009. 32795414 PMC7522766

[31] Krishn SR, Singh A, Bowler N, Duffy AN, Friedman A, Fedele C, Kurtoglu S, Tripathi SK, Wang K, Hawkins A, et al. Prostate cancer sheds the αvβ3 integrin in vivo through exosomes. Matrix Biol. 2019;77:41–57. doi:10.1016/j.matbio.2018.08.004. 30098419 PMC6541230

[32] Matoso A, Epstein JI. Grading of prostate cancer: past, present, and future. Curr Urol Rep. 2016;17(3):25. doi:10.1007/s11934-016-0576-4. 26874537

[33] Cheng L, Montironi R, Bostwick DG, Lopez-Beltran A, Berney DM. Staging of prostate cancer. Histopathology. 2012;60(1):87–117. doi:10.1111/j.1365-2559.2011.04025.x. 22212080

[34] Bery F, Cancel M, Chantôme A, Guibon R, Bruyère F, Rozet F, Mahéo K, Fromont G. The calcium-sensing receptor is a marker and potential driver of neuroendocrine differentiation in prostate cancer. Cancers (Basel). 2020;12(4):860. doi:10.3390/cancers12040860. 32252342 PMC7226072

[35] Yamada Y, Beltran H. Clinical and biological features of neuroendocrine prostate cancer. Curr Oncol Rep. 2021;23(2):15. doi:10.1007/s11912-020-01003-9. 33433737 PMC7990389

[36] Bhinder B, Ferguson A, Sigouros M, Uppal M, Elsaeed AG, Bareja R, Alnajar H, Eng KW, Conteduca V, Sboner A, et al. Immunogenomic landscape of neuroendocrine prostate cancer. Clin Cancer Res. 2023;29(15):2933–43. doi:10.1158/1078-0432.CCR-22-3743. 37223924 PMC10524949

[37] Saraswat M, Joenväära S, Musante L, Peltoniemi H, Holthofer H, Renkonen R. N-linked (N-) glycoproteomics of urinary exosomes [Corrected]. Mol Cell Proteomics: mCP. 2015;14(2):263–76. doi:10.1074/mcp.M114.040345. 25452312 PMC4350024

[38] Goetzl EJ, Kapogiannis D, Schwartz JB, Lobach IV, Goetzl L, Abner EL, Jicha GA, Karydas AM, Boxer A, Miller BL, et al. Decreased synaptic proteins in neuronal exosomes of frontotemporal dementia and Alzheimer’s disease. FASEB J. 2016;30(12):4141–48. doi:10.1096/fj.201600816R. 27601437 PMC5102122

[39] Yang J, Zhang Y, Gao X, Yuan Y, Zhao J, Zhou S, Wang H, Wang L, Xu G, Li X, et al. Plasma-derived exosomal ALIX as a novel biomarker for diagnosis and classification of pancreatic cancer. Front Oncol. 2021;11:628346. doi:10.3389/fonc.2021.628346. 34026608 PMC8131866

[40] Pietrowska M, Zebrowska A, Gawin M, Marczak L, Sharma P, Mondal S, Mika J, Polańska J, Ferrone S, Kirkwood JM, et al. Proteomic profile of melanoma cell-derived small extracellular vesicles in patients’ plasma: a potential correlate of melanoma progression. J Extracell Vesicles. 2021;10(4):e12063. doi:10.1002/jev2.12063. 33613873 PMC7876545

[41] Kim O, Hwangbo C, Tran PT, Lee JH. Syntenin-1-mediated small extracellular vesicles promotes cell growth, migration, and angiogenesis by increasing onco-miRnas secretion in lung cancer cells. Cell Death Dis. 2022;13(2):122. doi:10.1038/s41419-022-04594-2. 35136055 PMC8826407

[42] Folkmanis K, Junk E, Merdane E, Folkmane I, Folkmanis V, Ivanovs I, Eglitis J, Jakubovskis M, Laabs S, Isajevs S, et al. Clinicopathological significance of exosomal proteins CD9 and CD63 and DNA mismatch repair proteins in prostate adenocarcinoma and benign hyperplasia. Diagnostics (Basel). 2022;12(2):287. doi:10.3390/diagnostics12020287. 35204378 PMC8871402

[43] Franceschini GM, Quaini O, Mizuno K, Orlando F, Ciani Y, Ku S-Y, Sigouros M, Rothmann E, Alonso A, Benelli M, et al. Noninvasive detection of neuroendocrine prostate cancer through targeted cell-free DNA methylation. Cancer Discov. 2024;14:424–45. doi:10.1158/2159-8290.CD-23-0754. 38197680 PMC10905672

[44] Mugoni V, Ciani Y, Quaini O, Tomasini S, Notarangelo M, Vannuccini F, Marinelli A, Leonardi E, Pontalti S, Martinelli A. et al. Integrating extracellular vesicle and circulating cell-free DNA analysis using a single plasma aliquot improves the detection of HER2 positivity in breast cancer patients. J Extracell Biol. 2023;2(9):e108. doi:10.1002/jex2.108. 38046436 PMC10688391

[45] Colaprico A, Silva TC, Olsen C, Garofano L, Cava C, Garolini D, Sabedot TS, Malta TM, Pagnotta SM, Castiglioni I, et al. TCGAbiolinks: an R/Bioconductor package for integrative analysis of TCGA data. Nucleic Acids Res. 2016;44(8):e71. doi:10.1093/nar/gkv1507. 26704973 PMC4856967

[46] Robinson MD, McCarthy DJ, Smyth GK. edgeR: a bioconductor package for differential expression analysis of digital gene expression data. Bioinformatics. 2010;26(1):139–40. doi:10.1093/bioinformatics/btp616. 19910308 PMC2796818

[47] Benjamini Y, Hochberg Y. Controlling the false discovery rate - a practical and powerful approach to multiple testing. J Roy Stat Soc B Met. 1995;57(1):289–300. doi:10.1111/j.2517-6161.1995.tb02031.x.

[48] Beltran H, Prandi D, Mosquera JM, Benelli M, Puca L, Cyrta J, Marotz C, Giannopoulou E, Chakravarthi BVSK, Varambally S, et al. Divergent clonal evolution of castration-resistant neuroendocrine prostate cancer. Nat Med. 2016;22(3):298–305. doi:10.1038/nm.4045. 26855148 PMC4777652

[49] Salem I, Naranjo NM, Singh A, DeRita R, Krishn SR, Sirman LS, Quaglia F, Duffy A, Bowler N, Sayeed A, et al. Methods for extracellular vesicle isolation from cancer cells. Cancer Drug Resist. 2020;3:1–14. doi:10.20517/cdr.2019.118. 33062957 PMC7556721

[50] Testa A, Quaglia F, Naranjo NM, Verrillo CE, Shields CD, Lin S, Pickles MW, Hamza DF, Von Schalscha T, Cheresh DA, et al. Targeting the αVβ3/NgR2 pathway in neuroendocrine prostate cancer. Matrix Biol. 2023;124:49–62. doi:10.1016/j.matbio.2023.11.003. 37956856 PMC10823877

[51] Naranjo NM, Salem I, Harris MA, Languino LR. IFIT3 (Interferon Induced Protein with Tetratricopeptide Repeats 3) modulates STAT1 expression in small extracellular vesicles. Biochem J. 2021;478(21):3905–21. doi:10.1042/BCJ20210580. 34622927 PMC9121857

[52] Quaglia F, Krishn SR, Sossey-Alaoui K, Rana PS, Pluskota E, Park PH, Shields CD, Lin S, McCue P, Kossenkov AV, et al. The NOGO receptor NgR2, a novel αVβ3 integrin effector, induces neuroendocrine differentiation in prostate cancer. Sci Rep. 2022;12(1):18879. doi:10.1038/s41598-022-21711-5. 36344556 PMC9640716

